# A Systematic Review of Cost-Effectiveness Studies on Gastric Cancer Screening

**DOI:** 10.3390/cancers16132353

**Published:** 2024-06-27

**Authors:** Diedron Lewis, Laura Jimenez, Manel Haj Mansour, Susan Horton, William W. L. Wong

**Affiliations:** 1 School of Pharmacy, University of Waterloo, Waterloo, ON N2G 1C5, Canada; william.wl.wong@uwaterloo.ca; 2 Department of Community Health and Epidemiology, Dalhousie University, Halifax, NS B3H 4R2, Canada; laura.jimenez@dal.ca; 3 Department of Haematology and Oncology, Aga Khan University Hospital, Nairobi P.O. Box 30270-00100, Kenya; haj.manel@aku.edu; 4 School of Public Health Sciences, University of Waterloo, Waterloo, ON N2L 3G5, Canada; sehorton@uwaterloo.ca

**Keywords:** systematic review, gastric cancer screening, cost-effectiveness

## Abstract

**Simple Summary:**

This research set out to systematically review available cost-effectiveness studies on gastric cancer (GC) screening across the world. Of the studies reviewed, the majority were model-based, while fewer were prospective observational-based studies. The results of the review point to a distinction between Asian-based and non-Asian-based studies. The data revealed a higher risk of GC in Asian countries and their diasporas because of the elevated prevalence of one of the main risk factors within this population group, i.e., *Helicobacter pylori* (*Hp*) infections, compared with non-Asian populations. GC screening was mainly cost-effective in these high-risk groups, with a probability of at least 85% compared to no screening. Primary intervention, which involves *Hp* screening with eradication, was a preferred strategy as it addresses the main causative factor and limits the development of GC. Secondary intervention, which involves endoscopic screening, was also cost-effective but is typically used to identify adenocarcinomas rather than precancerous conditions. GC screening was generally not cost-effective among Western countries.

**Abstract:**

Gastric cancer (GC) poses notable economic and health burdens in settings where the incidence of disease is prevalent. Some countries have established early screening and treatment programs to address these challenges. The objectives of this systematic review were to summarize the cost-effectiveness of gastric cancer screening presented in the literature and to identify the critical factors that influence the cost-effectiveness of screening. This systematic review followed the Preferred Reporting Items for Systematic Reviews and Meta-analyses (PRISMA) guidelines. Economic evaluation studies of gastric cancer screening were reviewed from SCOPUS and PubMed. The Consolidated Health Economic Evaluation Reporting Standards 2022 (CHEERS 2022) was used to assess the quality of reporting presented in the selected articles. Only primary economic evaluation studies addressing the cost-effectiveness, cost–utility, and cost–benefit of gastric cancer screening were selected. Two reviewers scrutinized the selected articles (title, abstract, and full text) to determine suitability for the systematic review based on inclusion and exclusion criteria. Authors’ consensus was relied on where disagreements arose. The main outcome measures of concern in the systematic review were cost, effectiveness (as measured by either quality-adjusted life years (QALY) or life-years saved (LYS)), and incremental cost-effectiveness ratio (ICER) of screening versus either no screening or an alternative screening method. Thirty-one studies were selected for the final review. These studies investigated the cost-effectiveness of GC screening based on either primary, secondary, or a combination of primary and secondary interventions. The main primary intervention was *Helicobacter pylori* (*Hp*) screening with eradication, while the main secondary intervention was endoscopic screening. Cost-effectiveness was evaluated against no screening or screening using an alternative method in both observational and model-based studies. Screening was mainly cost-effective in Asian countries or their diasporas where the prevalence of GC was high. GC screening was generally not cost-effective among Western countries. GC screening can be cost-effective, but cost-effectiveness is dependent on context-specific factors, including geographical location, the prevalence of GC in the local population, and the screening tool adopted. However, there is benefit in targeting high-risk population groups in Asian countries and their diaspora for GC screening.

## 1. Introduction

Gastric cancer (GC) is one of the most common cancers in the world. In 2022, there were approximately one million newly diagnosed cases worldwide, the fifth highest among all newly diagnosed cases of cancer that year [1]. In terms of mortality, gastric cancer was also the fifth leading cause of cancer-related mortality globally, with approximately 659,936 deaths in 2022 [1]. New cases and deaths are disproportionately more prevalent among Asian countries and their diasporas, with China, Japan, India, and the Russian Federation accounting for approximately 60% of new cases globally in 2022 [1]. Similarly, these countries combined accounted for approximately 59% of total gastric cancer-related deaths across the world [1]. China alone accounted for approximately 37% and 40% of global GC cases and global GC deaths, respectively, in the same year [1].

Early screening, diagnosis, and treatment can potentially reduce the cost of care and notably improve clinical outcomes with a greater chance of survival and lower mortality, especially since early gastric cancer tends to be either asymptomatic or minimally symptomatic with epigastric discomfort and dyspepsia [2]. When detected at a later stage, the 5-year survival rate of gastric cancer is less than 30%, but early detection is associated with a 90% survival rate with appropriate treatment [3,4]. Treatment costs are exorbitant, and increasingly so with greater levels of intervention, posing a strain on limited healthcare resources [5]. In China, for example, the estimated cost of GC treatment was CNY 23.508 billion in 2018, representing the third-highest treatment cost among all types of cancers in that country [2]. Also, in the USA, the average monthly out-patient cost for GC patients was estimated to be between USD 34,002 and USD 72,778 (2017 USD) [5].

Generally, there are two approaches to the prevention of GC: primary prevention and secondary prevention methods. Primary prevention mainly involves *Helicobacter pylori* (*H. pylori*/*Hp*) screening and eradication, which potentially lowers GC incidence and mortality rates as the main risk factor is removed [6,7,8]. Meanwhile, secondary prevention commonly involves endoscopic screening (endoscopic screening here refers to gastroscopy/upper gastrointestinal endoscopy/esophagogastroduodenoscopy (EGD)) to detect GC, especially after gastric atrophy has been identified [9,10]. Other strategies along the continuum of screening tools include serological screening using pepsinogen, upper gastrointestinal series (UGI), tumor markers, microRNAs (miRNAs) in human serum, and Gastrin-17 [2,11,12].

Screening guidelines recommend a focus on high-risk populations, which typically includes those with high rates of *Hp* infections [2,13,14]. *Hp* is a group I carcinogen and known risk factor for the development of GC, particularly non-cardia gastric adenocarcinoma (NCGA), which is the most common type of GC [2,12,15,16,17]. In fact, up to 75% of all NCGA cases have an *H. pylori* histology [18] and approximately 50% of the Chinese population carries this infection with no visible symptoms [2]. Both gastric atrophy and intestinal metaplasia, which may stem from chronic inflammation caused by *Hp* infections, are also contributors to the development of GC [19,20,21]. Other notable risk factors for GC are smoking, diet, and genetics [12].

The Working Group of the International Agency for Research on Cancer (IARC) cautioned that GC will continue to be of global concern in the absence of effective control and prevention strategies [22]. This is even more likely in countries with higher incidence rates of *Hp* infections. The Working Group, however, acknowledged that with known interventions to prevent GC that target its risk factors, the burdens associated with the disease can be alleviated [22].

The objective of this study, therefore, is to perform a systematic review of the current literature on the cost-effectiveness of gastric cancer screening and to determine the key factors that impact cost-effectiveness. This review may prove useful to policy makers within and across both developed and developing countries to inform appropriate GC screening intervention strategies based on cost, effectiveness, and risk factors.

## 2. Materials and Methods

This systematic review followed the Preferred Reporting Items for Systematic Reviews and Meta-analyses (PRISMA) guidelines for reporting a systematic review [23]. The research protocol began with a broad search of the literature on health–economic evaluation studies on the cost-effectiveness of screening for esophageal, liver, pancreatic, and gastric cancers. However, for this systematic review, only gastric cancer is highlighted. The research protocol was registered on 14 October 2023 through PROSPERO, registration no. CRD42023467167.

SCOPUS and PubMed were the electronic databases used to perform the literature search. With the aid of two university-level health sciences librarians, search strategies were developed and tailored for each database. Key search terms included “stomach neoplasms”, “stomach cancer”, “gastric cancer”, “screening”, “early detection”, “economic evaluation”, and “cost-effectiveness analysis”. The literature search was restricted to studies published up to September 2023. The Supplementary Materials provide the complete search strategy along with mesh combinations for each database. (see the Supplementary Material File S1).

COVIDENCE was used as a repository for all articles identified from the initial literature search. This was also where the PRISMA flow chart was generated. The literature search was not restricted to any particular country or group of countries. The authors recognized that the cancer of concern in this review is less prevalent in North America and Europe and more prevalent in Southeast Asia.

The specific inclusion criteria were as follows:

1. Studies that focused on the screening of stomach cancers through primary and/or secondary methods compared to no screening or screening using alternative methods;
2. Studies that focused on populations at average risk or above average risk for gastric cancer;
3. Studies that reported on patient outcomes measured in terms of quality-adjusted life-years (QALY) or life-years gained (LYG);
4. Studies based on decision-analytic modeling assessing both the long-term effectiveness and the cost-effectiveness of different early detection;
5. Studies that reported the incremental cost-effectiveness ratio (ICER) or provided data for ICER calculation;
6. Studies that outlined cost per quality-adjusted life-year (QALY) or cost per life-year gained, or cost per utility gained;
7. Studies that had full economic evaluations;
8. Studies published in English.

The exclusion criteria were as follows:

1. Non-original studies;
2. Studies not published in English;
3. Grey literature;
4. Systematic reviews, editorials, letters, abstracts, and studies that are not full health economic evaluations or evaluated only follow-up or treatment strategies.

**Figure 1:**
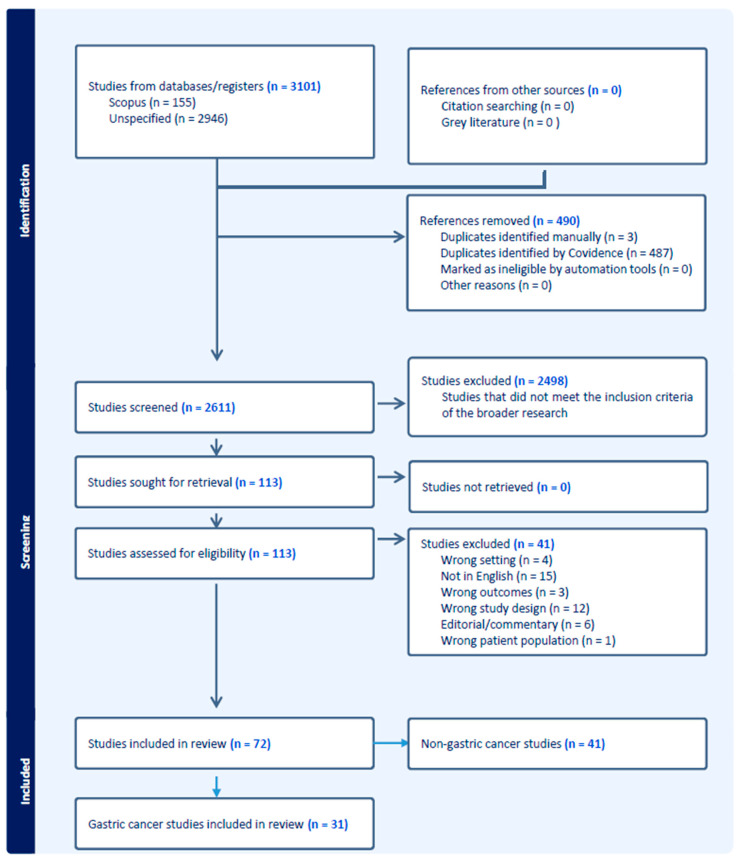
PRISMA diagram-criteria [23].

In selecting the final articles for the systematic review, firstly, two authors (L.J. and D.L.) completed title–abstract screening of all the studies generated from the literature search in SCOPUS and PubMed. This screening process was guided by the inclusion and exclusion criteria described above and presented in Figure 1. Authors’ consensus was relied on where disagreements arose.

Following the selection of relevant articles through the initial screening process, a full-text review was conducted to extract all pertinent pieces of data from each of the remaining articles. The reviewers applied the inclusion and exclusion criteria to form the final list of studies.

From the final list of studies, the Consolidated Health Economic Evaluation Reporting Standards 2022 (CHEERS 2022) [24] was used to assess the adequacy/quality of reporting of each study. The following information were also extracted from each study: study settings, target populations, study objectives, screening strategies, economic evaluation model and its features (namely, model type, study perspective, discount rate, time horizon, sensitivity analysis, and willingness to pay (WTP) threshold), clinical outcomes, costs associated with screening, and study recommendations. The main outcome indicators were quality-adjusted life years (QALY), life-years saved (LYs), incremental cost-effectiveness ratio (ICER), and incremental cost–utility ratio (ICUR).

## 3. Results

Thirty-one studies were ultimately selected for this review based on the inclusion–exclusion criteria. This selection process is illustrated in the PRISMA diagram, Figure 1. Table 1, Table 2 and Table 3 provide a summary of the data extracted from the selected studies. Notable variations across the selected studies can be identified, particularly with respect to country setting, target population, time horizon, discount rate, study perspective, study objective, age range, cancer risk, screening compliance, screening strategies, and willingness to pay threshold.

Generally, studies conducted economic evaluations of GC screening methods using cost-effectiveness analyses that report on the ratios (ICERs) as the principal outcome measure. ICER was typically measured as cost per quality-adjusted life year (QALY), except for a few studies that estimated cost per life-year saved (LYS) [14,25,26,27]. Cho et al. (2013) estimated cost per survival, while Wei et al. (2011) performed a cost–benefit analysis [28,29]. Economic evaluations were conducted from several perspectives, namely, societal, healthcare system, healthcare payer, third party payer, ministry of health, healthcare provider, and public healthcare.

**Table 1:** Characteristics of included studies.

References	Country/Region	Cancer Risk	Intervention Strategy	Reference Strategy	Study Objective
Zhou et al., 2011 [30]	Zhuanghe County, China	High risk	Epidemiological survey, serum PG + endoscopy, pathological examination	No screening	To evaluate the economic cost-effectiveness of the screening program for gastric cancer in a high-risk population
Zheng & Liu, 2023 [2]	China	High risk	*Hp* screening, NGCS	No screening	To evaluate the cost-effectiveness of *Hp* and new gastric cancer screening scoring system in areas with a high incidence of gastric cancer
Yeh et al., 2016 [12]	USA	Current smokers, former smokers, non-smokers	Serum PG, endoscopic-based screening, *Hp* screening	No screening	To estimate the cost-effectiveness of non-cardia gastric adenocarcinoma (NCGA) screening strategies based on new biomarker and endoscopic technologies
Xia et al., 2021 [13]	China	High risk	Endoscopic screening: once per lifetime, and every 10 years, 5 years, 3 years, 2 years	No screening	To evaluate the cost-effectiveness of endoscopic screening for esophageal and gastric cancers among people aged 40–69 years in areas of China where risk of these cancers is high
Wu et al., 2016 [31]	Singapore	Intermediate risk	Annual EGD surveillance; 2-yearly EGD screenings	No EGD follow-up	To identify the optimal strategy in the prevention of GC in Singapore based on cost-effectiveness ratios estimated by Markov models
Wei et al., 2011 [28]	Linzhou, China	High risk	Endoscopy with mucosal iodine staining in combination with index biopsies	Not reported	To evaluate the cost–benefit of the Early Detection and Early Treatment of Esophageal and Cardiac Cancer (EDETEC) program
Wang et al., 2022 [32]	China	Average and high risk	*Hp* eradication; electronic endoscopy (one-time, annual, biennial, or triennial)	Status quo	To compare the effects, affordability, and cost-effectiveness of different GC prevention approaches
Tsuji et al., 1991 [27]	Japan	Not reported	Gastro-fluorography screening	No screening	To compare the CE ratio of colorectal cancer screening with other cancer screening program in Japan (gastric cancer)
Suh et al., 2020 [25]	Republic of Korea	High risk	Screening: endoscopy or UGI	No screening	To evaluate the treatment benefit and cost of the National Cancer Screening Program for gastric cancer
Shah et al., 2020 [15]	USA	Average risk and met colorectal cancer screening requirement	1-time EGD during colonoscopy + EDG every 3 years if IM is identified; 1-time EGD during colonoscopy + biennial EGD regardless of finding	No screening	To compare the CE of two endoscopic strategies for gastric cancer screening with no screening (by sex and ethnic group)
Saumoy et al., 2018 [33]	USA	High risk	1-time EGD during colonoscopy + EDG every 3 years if IM is identified; 1-time EGD during colonoscopy + biennial EGD regardless of finding	No screening	To determine whether selected non-cardia gastric cancer screening for high-risk races and ethnicities within the US is cost-effective
Saito et al., 2018 [34]	Japan	High risk	ABC method + endoscopy if needed	Annual endoscopic screening	To evaluate the cost-effectiveness of gastric cancer screening by the ABC method in Japanese individuals from the perspective of the Japanese healthcare payer
Qin et al., 2022 [35]	China	High risk	7 GCRSS strategies with different start ages: 40, 45, 50, 55, 60, 65, and 70 years	No screening	To assess the clinical benefits, harm, cost, and cost-effectiveness of the gastric cancer risk score scale (GCRSS) screening strategy from a Chinese healthcare system perspective
Qin et al., 2022 [36]	China	High risk	30 alternative screening strategies with varying starting ages, including NGCS, modified NGCS, and endoscopy with varying screening intervals	No screening	To assess the clinical benefits and the cost-effectiveness of the NGCS strategy in GC high-risk areas of China from a societal perspective
Oh et al., 2022 [37]	USA	Average risk	1. Single-population screening for *H. pylori* using 13C-UBT and treating those who tested positive with eradication therapy, and 2. single-population screening for *H. pylori* with upper endoscopy and PCR of gastric biopsies and treating those who tested positive with eradication therapy	No screening with opportunistic eradication	To evaluate the cost-effectiveness of C-UBT and PCR population screening strategies of *H. pylori* for the prevention of PUD and gastric cancer in the United States
Ma et al., 2022 [38]	China	High risk	1. FBCM; 2. screen and treat	No screening	To investigate the cost-effectiveness of family-based *Hp* infection control and management (FBCM)
Li et al., 2014 [39]	Zhuanghe County, China	High risk	Gastric cancer screening program: epidemiology survey, serum PG, health education, gastroscope, gastric mucosa biopsy	Not reported	To assess the screening program on gastric cancer in Zhuanghe, China, using the health economics methodology
Kowada, 2023 [40]	Japan	Average risk	Annual, biennial, and triennial endoscopic screening and *Hp* eradication strategy	No screening	To evaluate which strategy was the most optimal and cost-effective among the *Hp* eradication strategy; annual, biennial, and triennial endoscopic screening; and no screening as a national gastric cancer prevention program
Kowada, 2021 [41]	Japan	Not reported	Annual and biennial endoscopic screening after *Hp* eradication strategy	No screening	To assess the cost-effectiveness of annual endoscopy versus biennial endoscopy versus no screening for gastric cancer screening in patients after successful *Helicobacter pylori* eradication
Kowada, 2019 [42]	Japan	High risk	1. UGI; 2. endoscopy	*Hp* screening	To assess the cost-effectiveness of *H. pylori* screening compared to UGI and endoscopy to evaluate the optimal gastric cancer screening method in high-prevalence countries
Kapoor et al., 2020 [43]	Singapore	Intermediate risk	Initial screening using miRNA test followed by endoscopy for test-positive individuals and a 3-yearly follow-up screening for test-negative individuals	No screening	To evaluate the cost-effectiveness of a novel screening strategy using a microRNA (miRNA) blood test as a screen, followed by endoscopy for diagnosis confirmation in a 3-yearly population screening program for gastric cancer
Huang et al., 2020 [44]	Japan	Average risk	14 endoscopic screening scenarios with various starting ages, stopping ages, and screening intervals	No screening	To estimate the cost-effectiveness of current screening guidelines and alternative screening strategies in Japan
Gupta et al., 2011 [45]	USA	Average risk	Endoscopy with colonoscopy	No endoscopy during colonoscopy	To evaluate the cost-effectiveness of screening the general population for upper gastrointestinal cancer by performing an upper endoscopy at the time of screening colonoscopy
Enriquez-Sanchez et al., 2022 [46]	Mexico	Intermediate risk	EGD± follow-up, EGD every 2 years, serum PG detection	No screening	To determine the cost and benefit of esophagogastroduodenoscopy, serum pepsinogen detection, and no screening
Di Giulio et al., 2009 [26]	USA	Not reported	Endoscopy	No screening	To assess the clinical and economic impact of ASGE and EPAGE guidelines in selecting patients referred for upper endoscopy (EDG) relative to the direction of gastro-oesophageal cancer
Dan et al., 2006 [47]	Singapore	Intermediate risk and various high-risk groups	2 yearly endoscopies	No screening	To conduct a cost–utility analysis to determine whether endoscopic screening for stomach cancer in an intermediate-risk population would be cost-effective and to better define the high-risk groups in the population who would benefit from such a strategy
Cho et al., 2013 [29]	Republic of Korea	Not reported	1. Upper-gastrointestinal X-ray; 2. endoscopy	No screening	To evaluate the cost-effectiveness outcomes of the national cancer screening program for gastric cancer (UGI and endoscopy)
Chang et al., 2012 [48]	Republic of Korea	Not reported	1. Endoscopy; 2. UGI series	No screening	To explore the cost-effectiveness of various gastric cancer screening programs using endoscopy or UGI series in Korea relative to no screening and determine the most favorable screening alternative for gastric cancer with regard to starting age and screening interval
Babazono & Hillman, 1995 [14]	Japan	High risk	1. Yearly indirect X-ray, followed by endoscopy if positive; 2. yearly direct X-ray, followed by endoscopy if positive; and 3. yearly endoscopy alone	No screening	The specific aims of this case study of gastric cancer screening in Japan were to (a) determine the most cost-effective strategy; (b) decide on the optimal target ages stratified by sex; and (c) identify the change of cost-effectiveness between the 1980s and 1990s
Ascherman & Hur, 2021 [49]	Brazil, France, Japan, Nigeria, and the United States	High risk	Endoscopic screening: once per lifetime, and every 10 years, 5 years, 2 years	No screening	To performance a cost-effectiveness analysis to compare screening and surveillance strategies for gastric cancer in Brazil, France, Japan, Nigeria, and the United States
Areia et al., 2018 [50]	Portugal	Intermediate risk	1. Stand-alone upper endoscopy, 2. endoscopy combined with a colorectal cancer screening colonoscopy after a positive fecal occult blood test or pepsinogen serologic screening	No Screening	To determine the cost–utility of screening strategies for gastric cancer in a European country

NGCS—new gastric cancer screening scoring system; Serum PG—serum pepsinogen; GCRSS—gastric cancer risk score scale; NCGA—non-cardia gastric adenocarcinoma; EGD—esophagogastroduodenoscopy; GC—gastric cancer; UGI—upper gastrointestinal series; ABC method—a combination of serum *Helicobacter pylori* IgG antibody (HPA) and pepsinogen assays; CE—cost-effective; IM—intestinal metaplasia; FBCM—family-based *H. pylori* infection control and management; C-UBT—carbon urea breath test; PCR—polymerase chain reaction; ASGE—American Society for Gastrointestinal Endoscopy; EPAGE—European Panel on the Appropriateness of Gastrointestinal Endoscopy; PUD—peptic ulcer disease; miRNA—microRNA.

**Table 2:** Summary of study methods of included studies.

References	Age Range	Analytical Model	Cycle Length	Time Horizon	Compliance	Perspective	Discount Rate	Source of Clinical Input
Zhou et al., 2011 [30]	>35 years	Observational	Not applicable	2 years	Full	Direct cost	5%	Prospective data from study population
Zheng & Liu, 2023 [2]	40–77 years	Markov	1 year	37 years	Full	Not reported	3%	Literature
Yeh et al., 2016 [12]	20–death	Markov	Monthly	Lifetime	Full	Societal	3%	Literature, SEER
Xia et al., 2021 [13]	40–69 years	Markov	1 year	Lifetime	49%	Healthcare system	5%	Clinical trial, literature
Wu et al., 2016 [31]	50–69 years	Markov	1 year	Lifetime	Full	Healthcare payer	3%	Database, literature
Wei et al., 2011 [28]	40–69 years	Observational	Not applicable	3 years	67%	3rd-party payer	Not reported	Prospective data from study population
Wang et al., 2022 [32]	40–65 years	Markov	1 year	15 years	89%	Not reported	3%	Database, literature
Tsuji et al., 1991 [27]	40–79 years	Simulation Model	Not reported	Not reported	Not reported	Not reported	Not reported	Literature, databases
Suh et al., 2020 [25]	≥40 years	Observational	Not reported	10 years	Not reported	Ministry of Health: National Health Insurance	Not reported	National Health Insurance Service, Korean National Health Insurance Big Data Base
Shah et al., 2020 [15]	50–80 years	Markov	1 year	30 years	Not reported	Healthcare system	3%	SEER, registries, literature
Saumoy et al., 2018 [33]	50–80 years	Markov	1 year	30 years	Not reported	Healthcare system	3%	Literature, CDC
Saito et al., 2018 [34]	50–80 years	Markov	1 year	30 years	60%	Healthcare payer	2%	Literature, registry
Qin et al., 2022 [35]	40–70 years	Markov	1 year	Lifetime	Full	Healthcare system	5%	Literature, registries
Qin et al., 2022 [36]	40–70	Markov	1 year	Lifetime	Full	Societal	5%	Literature, registries
Oh et al., 2022 [37]	40–100 years	Markov	1 year	Lifetime	Full	3rd- party payer	3%	Literature, registries
Ma et al., 2022 [38]	18–78 years	Markov	1 year	Lifetime	Not reported	Healthcare provider	3%	Database, literature
Li et al., 2014 [39]	40–69 years	Observational	Not reported	2008–2012	Full	Not reported	3%	Program data
Kowada, 2023 [40]	20–80 years	Markov	1 year	Lifetime	49.50%	Healthcare payer	3%	Literature, registries
Kowada, 2021 [41]	50–lifetime	Markov	1 year	Lifetime	Not reported	Healthcare payer	3%	Literature, registries
Kowada, 2019 [42]	40–80 years	Markov	1 year	Lifetime	Not reported	Healthcare payer	3%	Registries, literature
Kapoor et al., 2020 [43]	50–75 years	Markov	1 year	25 years	45%	Healthcare	3%	Literature, registries
Huang et al., 2020 [44]	40–80 years	Markov	1 year	Lifetime	Not reported	Societal	3%	Literature, registries
Gupta et al., 2011 [45]	50-year-olds	Markov	1 year	30 years	Full	3rd-party payer	3%	Registries
Enriquez-Sanchez et al., 2022 [46]	50–80 years	Markov	1 year	30 years	Not reported	Public healthcare	3%	Literature, registries
Di Giulio et al., 2009 [26]	60-year-olds	Decision Tree	Not applicable	Lifetime	Not reported	Societal	Not reported	Registries
Dan et al., 2006 [47]	50–70 years	Markov	1 year	Lifetime	Full	Societal	3%	Literature, registries
Cho et al., 2013 [29]	40 years+	Observational	Not reported	9 years	Varying	Direct + indirect + productivity loss	Not reported	Registries
Chang et al., 2012 [48]	50–80 years	Markov	1 year	Lifetime	Male:18.7%; Female: 24.7%	Societal	3%	Literature, registries
Babazono & Hillman, 1995 [14]	40–80 years	Markov	1 year	10 years	13%	Government payer	5%	Literature, registries
Ascherman & Hur, 2021 [49]	40–75 years	Markov	1 year	35 years	Full	Not reported	3%	Literature, SEER, registries
Areia et al., 2018 [50]	50–75 years	Markov	1 year	25 years	Not reported	Not reported	3%	Literature, SEER, registries

CDC—Centers for Disease Control and Prevention; SEER—Surveillance, Epidemiology, and End Results.

**Table 3:** Summary of outcomes of included studies.

References	Intervention Strategy	Reference Strategy	Currency/ Year	Incremental Cost-Effectiveness Ratio (ICER)	Willingness to Pay Threshold	Sensitivity Analysis	Sensitive Variables—1-Way SA	Probability Sensitivity Analysis	CHEERS Checklist
Zhou et al., 2011 [30]	Epidemiological survey, serum PG + endoscopy, pathological examination	No screening	2001	USD 459/QALY	Not reported	Not reported	Not reported	Not reported	Not reported
Zheng & Liu, 2023 [2]	*Hp* screening, NGCS	No screening	CNY	No screening: dominated to NGCS; NGCS to *Hp* screening: CNY 538,680/QALY; *Hp* screening to no screening: CNY 5536/QALY	CNY 80,976/QALY	1-way SA and PSA	NGCS: probability of transitioning from IM to dysplasia; *Hp* screening: probability of transitioning from *Hp* gastritis to AG	NGCS CE 95%; *Hp* CE 99%	Not reported
Yeh et al., 2016 [12]	Serum PG, endoscopic-based screening, *Hp* screening	No screening	2012 USD	*Hp* and endoscopic screening dominated. Serum PG: USD 105,400/QALY	USD 100,000/QALY	1-way SA & PSA	Serum PG: *Hp* prevalence, screen age, serum PG test sensitivity, endoscopic follow-up costs	Serum PG CE 47% for general population; 97% for current smokers; 85% former smokers	Not reported
Xia et al., 2021 [13]	Endoscopic screening: once per lifetime, and every 10 years, 5 years, 3 years, 2 years	No screening	2019 USD	(Screen age 40–45 years) vs. no screening—ICER: once: USD 3035, 10 years: USD 1566; 5 yrs: USD 1781; 3 years: USD 2720; 2 yrs: USD 4511	WHO definition: not CE if ICER > 3times GDP per capita (USD 10,276)	1-way SA & PSA	Varying utility scores for GC and EC	Screening every 2 years CE 98% compared to other strategies	Reported but not included
Wu et al., 2016 [31]	Annual EGD surveillance; 2-yearly EGD screening	No EGD follow-up	2011 SGD	SGD 23,470–SGD 39,761/QALY	SGD 44,000/QALY	1-way SA & PSA	Discount rate, surveillance start age, cost of program, cost of EGD/biopsy, odds of precancerous legions, utility of GC stage 1	Surveillance CE 96.5%	Not reported
Wei et al., 2011 [28]	Endoscopy with mucosal iodine staining in combination with index biopsies	Not reported	CNY	Benefit–cost ratio: 4.49–10.37	Not reported	Not reported	Not reported	Not reported	Not reported
Wang et al., 2022 [32]	*Hp* eradication; electronic endoscopy (one-time, annual, biennial, or triennial)	Status quo	2020 CNY	*Hp* dominant	CNY 70,000/ QALY	1-way SA	GC prevalence, annual progress rates between the states AG, IN, ESC, and ASC; the compliance rate with and success rate of *Hp* treatment as well as eradication-related impact on state transition; and the prevalence of risk factor(s) used to define the target population were the parameters with the most marked impact on cost-effectiveness among the strategies	Not reported	Reported but not included
Tsuji et al., 1991 [27]	Gastro-fluorography screening	No screening	JPY	Male: JPY 606.3K/LYS; female: JPY 1542.5K/LYS	Not reported	Not reported	Not reported	Not reported	Not reported
Suh et al., 2020 [25]	Screening: endoscopy or UGI	No screening	USD/ KRW	Average cost/LYS: USD 20,309	WHO definition: not CE if ICER > 3 times GDP per capita (USD 20,565)	Not reported	Not reported	Not reported	Not reported
Shah et al., 2020 [15]	1-time EGD during colonoscopy + EDG every 3 years if IM is identified; 1-time EGD during colonoscopy + biennial EGD regardless of finding	No screening	2015 USD	1-time: USD 74,329/QALY; biennial: absolutely dominated	USD 100,000/ QALY	1-way SA and PSA	Transition probability: local–regional NCGA, dysplasia–local, regional–distant; probability that dysplastic lesion undergo endoscopy, cost of endoscopy	1-time endoscopy is preferred at the WTP	Not reported
Saumoy et al., 2018 [33]	1-time EGD during colonoscopy + EDG every 3 years if IM is identified; 1-time EGD during colonoscopy + biennial EGD regardless of finding	No screening	2015 USD	1-time: USD 71,451–USD 80,278/QALY; for non-Hispanic whites, 1-time is not CE; biennial: dominated in all scenarios	USD 100,000/QALY	1-way SA and PSA	Transition probability: IM–dysplasia, dysplasia–local, local–regional; probability of IM, cost of upper endoscopy, EGD, gastrectomy	No screening is CE for non-Hispanic whites 56.2%; 1-time endoscopy CE 55.2% (Hispanic), 52.9% (non-Hispanic blacks), 58.9% (Asians)	Not reported
Saito et al., 2018 [34]	ABC method + endoscopy if needed	Annual endoscopic screening	2014 USD	ABC dominant: more effective and less costly	USD 50,000/ QALY	1-way SA and PSA	ABC remained CE for all range of values	ABC CE 99.7% at WTP USD 10,000/QALY	Not reported
Qin et al., 2022 [35]	7 GCRSS strategies with different start ages: 40, 45, 50, 55, 60, 65, and 70 years	No screening	2021 USD	USD 10,315–USD 27,446/QALY	WHO definition: not CE if ICER > 3 times GDP per capita (USD 37,655 per QALY)	1-way SA and PSA	Risk of progressing dysplasia after surgery; cost of surgery	40-GCRSS CE 85.6%	Not reported
Qin et al., 2022 [36]	30 alternative screening strategies with varying starting ages, including NGCS, modified NGCS and endoscopy with varying screening intervals	No screening	2021 USD	NGCS strategies: USD 12,514–USD 118,852/QALY. 40-NGCS: USD 15,668/QALY. 40-NGCS is the most optimal screening strategy.	1.45 times GDP per capita: USD 17,922/QALY	1-way SA and PSA	Relative risk of progressing to preclinical stage I after surgery, transition probability from gastritis to atrophy, and cost of surgery	40-NGCS CE 86.3%	Not reported
Oh et al., 2022 [37]	1. Single population screening for *H. pylori* using 13C-UBT and treating those who tested positive with eradication therapy, and 2. single population screening for *H. pylori* with upper endoscopy and PCR of gastric biopsies and treating those who tested positive with eradication therapy	No screening with opportunistic eradication	2020 USD	Versus no screening: C-UBT: USD 116.46; PCR + biopsy: USD 2329.69. C-UBT vs. PCR: USD 38,591.89	USD 100,000/ QALY	1-way SA and PSA	Risk of gastric cancer if *H. pylori*-positive, the risk of gastric cancer after *Hp* eradication, *H. pylori* prevalence, and costs of screening	PCR CE 65%	Reported
Ma et al., 2022 [38]	1. FBCM; 2. screen and treat	No screening	2020 USD	Versus no screening: FBCM: USD 9.18/QALY; screen and treat: USD 12.08/QALY; screen and treat vs. FBCM: USD 27.44/QALY	USD 31,315/QALY; 3 times GDP per capita	1-way SA and PSA	Variables not sensitive	Screen and treat CE > 99%	Not reported
Li et al., 2014 [39]	Gastric cancer screening program: epidemiology survey, serum PG, health education, gastroscope, gastric mucosa biopsy	Not reported	Not provided	CNY 1370/QALY	Not reported	1-way SA on discount rate	CNY 1413/QALY for discount rate of 5%	Not reported	Not reported
Kowada, 2023 [40]	Annual, biennial, and triennial endoscopic screening and *Hp* eradication strategy	No screening	2021 USD	USD 24.4/QALY	USD 50,000/ QALY	1-way SA and PSA	ICER not sensitive to selected variables	*Hp* eradication CE 100%	Not reported
Kowada, 2021 [41]	Annual and biennial endoscopic screening after *Hp* eradication strategy	No screening	2018 USD	Biennial: USD 135,566/QALY	USD 100,000/ QALY	1-way SA and PSA	Incidence of gastric cancer and the proportion of stage I	Biennial CE 100%	Not reported
Kowada, 2019 [42]	1. UGI; 2. endoscopy	*Hp* screening	2018 USD	*Hp* screening dominated all other options	USD 50,000/ QALY	1-way SA, multiway sensitivity analysis, and PSA	Results robust	*Hp* screening CE 100%	Not reported
Kapoor et al., 2020 [43]	Initial screening using miRNA test followed by endoscopy for test-positive individuals and a 3-yearly follow-up screening for test negative individuals	No screening	2018 USD	Intervention: USD 40,971/QALY	USD 70,000/ QALY	1-way SA and PSA	Incidence of gastric cancer, cost of screening tests, sensitivity and specificity of the miRNA test, and endoscopy and utility values of cancer-free individuals	Intervention CE 95%	Not reported
Huang et al., 2020 [44]	14 endoscopic screening scenarios with various starting ages, stopping ages, and screening intervals	No screening	2015 USD	Triennial screening of 50–75 yr olds: ICER: USD 45,665/QALY	USD 50,000/ QALY	1-way SA and PSA	Direct cost of endoscopy, direct cost of EGD, sensitivity of endoscopy, complete resection rate of EGD, specificity of endoscopy, first-year medical cost for local cancer, and first-year medical cost for distal cancer and regional cancer	Intervention CE 92.6%	Reported
Gupta et al., 2011 [45]	Endoscopy with colonoscopy	No endoscopy during colonoscopy	2009 USD	USD 115,664/QALY	USD 50,000/ QALY	1-way SA	Prevalence rate of gastric cancer	Not reported	Not reported
Enriquez-Sanchez et al., 2022 [46]	EGD± follow-up, EGD every 2 years, serum PG detection	No screening	2019 USD	EGD ± follow-up: USD 129/QALY; serum PG: USD 1590/QALY	USD 9000/ QALY	PSA	Not reported	EDG± follow-up CE 100%	Not reported
Di Giulio et al., 2009 [26]	Endoscopy	No screening	2007 USD	Appropriate endoscopy: USD 16,577/LYG; inappropriate endoscopy: USD 301,203/LYG	USD 150,000/ LYG	Systematic analysis	Cancer prevalence	Not reported	Not reported
Dan et al., 2006 [47]	2 yearly endoscopies	No screening	2003 USD	USD 26,836/QALY	USD 28,000	1- and 2-way SA	Cost of screening endoscopy and the distribution of cancer stage at screening	Not reported	Not reported
Cho et al., 2013 [29]	1. Upper-gastrointestinal X-ray; 2. endoscopy	No screening	2009 KRW	UGI: KRW 260,201–371,011,000 KW/survival; endoscopy: KRW 119,099,000–17,870,000 KW/survival	Not reported	Scenario analysis	Upper screening age limit	Not reported	Not reported
Chang et al., 2012 [48]	1. Endoscopy; 2. UGI series	No screening	2008 USD	Male: USD 4820/QALY, female: USD 6073/QALY	WHO definition: not CE if ICER > 3 times GDP per capita (USD 19,162)	1-way SA	Screening cost, distribution of cancer stages at screening	Not reported	Not reported
Babazono & Hillman, 1995 [14]	1. Yearly indirect X-ray, followed by endoscopy if positive; 2. yearly direct X-ray, followed by endoscopy if positive; and 3. yearly endoscopy alone	No screening	1990 USD	Male: USD 50,888–USD 6376; female: USD 40,568–USD 19,424	Not reported	1-way SA and scenario analysis	No change in conclusion	Not reported	Not reported
Ascherman & Hur, 2021 [49]	Endoscopic screening: once per lifetime, and every 10 years, 5 years, 2 years	No screening	2020 USD	Screening every 10 years most CE but does not meet WTP threshold. Japan: USD $76,221/QALY	2 times GNI per capita. Brazil: USD 18,822; France: USD 84,596; Japan: USD 85,069; Nigeria: USD 4036; US: USD 129,900	1-way SA	Starting age of screening, cost of endoscopy, and baseline probability of local gastric cancer at time of diagnosis	Not reported	No reported
Areia et al., 2018 [50]	1. Stand-alone upper endoscopy, 2. endoscopy combined with a colorectal cancer screening colonoscopy after a positive fecal occult blood test or pepsinogen serologic screening	No Screening	2016 EUR	Endoscopy: EUR 15,407/QALY; endoscopy + colonoscopy: EUR 30,908/QALY; serum PG: EUR 143,344/QALY	2-times 2016 GNI per capita. EUR 37,000/QALY	1-way SA and PSA	Endoscopy costs, the number of endoscopies per patient over the screening age range and the ASR of gastric cancer	Intervention CE 86%	Reported but not included

NGCS—new gastric cancer screening scoring system; Serum PG—serum pepsinogen; EGD—esophagogastroduodenoscopy; UGI—upper gastrointestinal series; ABC method—a combination of serum *Helicobacter pylori* IgG antibody (HPA) and pepsinogen assays; GCRSS—gastric cancer risk score scale; FBCM—family-based *H. pylori* infection control and management; CE—cost-effective; ICER—incremental cost-effectiveness ratio; WHO—World Health Organization; GDP—gross domestic product; GC—gastric cancer; LYS—life-year saved; C-UBT—carbon urea breath test; QALY—quality-adjusted life year; WTP—willingness to pay; PCR—polymerase chain reaction; NCGA—non-cardia gastric adenocarcinoma; 1-way SA—one-way sensitivity analysis; PSA—probabilistic sensitivity analysis; IM—intestinal metaplasia; AG—atrophic gastritis; EC—esophageal cancer; IN—intraepithelial neoplasia; ESC—early-stage cancer; ASC—advanced-stage cancer; ASR—age-standardized rate; miRNA—microRNA.

### 3.1. Interventions Identified from the Systematic Review

The studies identified in this systematic review either focused on primary, secondary, or both primary and secondary intervention strategies. The most common primary interventions sought to identify GC risk factors like *Hp* infection, gastric atrophy, intestinal metaplasia, and dysplasia using either, or a combination of, *Helicobacter pylori* IgG antibody (HPA), 14C-urea breath test (UBT), Gastrin-17 (G-17), MiRNA screening, or serum pepsinogen (PG) I and I/II (pepsinogen levels at or below 70 mg L^−1^ (≤70 μg/L) for pepsinogen I and 3.0 for the pepsinogen ratio I/II warrant follow-up gastric cancer screening and diagnosis [12]). Some studies even developed a scoring method to determine the risk of developing GC based on combinations of the aforementioned indicators. In fact, two studies investigated the cost-effectiveness of the new gastric cancer screening scoring system (NGCS) in China, which is a scoring system that assesses a person’s status with respect to *Hp* infection, PG levels, G-17 levels, age, and sex in order to determine GC risk levels where risk is classified as high, moderate, or low [2,30]. In a similar study, the gastric cancer risk score scale (GCRSS) was evaluated—this however determined risk based on *Hp* infection, PG levels, and G-17 levels [31]. Another study focused on the “ABC method”, which combines both *Hp* and PG screening to assess GC risk [32].

Endoscopy, particularly, gastrointestinal endoscopy, otherwise known as gastroscopy or esophagogastroduodenoscopy, was identified as the main secondary intervention among the studies, albeit at different frequencies, including one-time, annual, biennial, triennial, every 5 years, and every 10 years. Other radiographic procedures like upper gastro-intestinal series (UGI) were less commonly considered [14,29,33,34].

The aforementioned interventions were compared to no screening in most cases and, in some cases, against an alternative strategy: either the next most effective screening strategy or a previous less costly strategy.

There was general consensus across the studies on the outcomes of the economic evaluations. Studies that focused on primary interventions found these interventions to generally be cost-effective compared to no screening in the prevention of GC, with a probability of at least 85% (across various study perspectives and willingness to pay (WTP) thresholds) [2,11,12,30,31,32,35,36]. These primary interventions range from *Hp* screening and treatment only to composite risk measurement tools like NGCS, ABC, and GCRSS. Even further, studies that compared both primary and secondary interventions demonstrated that primary interventions involving *Hp* screening were more cost-effective at least 85% of the times [11,30,31,32,33,35,37,38]. Four of these studies were based on the Japanese population [32,33,37,38], another three were based on the Chinese population [11,30,31], and one was based on the Mexican population [35]. In contrast, one study on a US-based population did not find population-wide *Hp* screening to be cost-effective at a WTP of USD 100,000/QALY, although cost-effectiveness was more likely (85–97%) among former and current smokers using serum pepsinogen in the USA from a societal perspective compared to no screening [12].

For studies that investigated only secondary interventions, endoscopic screening was found to be cost-effective, particularly in high-risk regions, with a probability between 92 and 100% compared to no screening [13,38,39,40,41]. In regions with intermediate risk, like Europe and the USA, endoscopic screening was cost-effective among high-risk ethnic groups and when performed during a colonoscopy (ICER: USD 71,451–USD 80,278/QALY (2015 USD) at a WTP of USD 100,000/QALY in the USA and ICER: EUR 15,407/QALY (2016 EUR) at a WTP of EUR 37,000/QALY in Portugal [15,42,43]. See Table 1, Table 2 and Table 3.

### 3.2. Cost-Effectiveness Analysis of Asian-Based Versus Non-Asian-Based Studies

The studies selected for the systematic review were grouped into one of two categories based on geographical location of each respective study population. These categories are Asian-based studies and non-Asian-based studies.

Among Japanese-based studies, primary intervention, in particular, *Hp* screening and treatment, was found to be cost-effective compared to either no screening or endoscopic screening 100% of the times at a WTP threshold of USD 50,000/QALY from a healthcare payer perspective [33,37]. In one Chinese-based study, *Hp* was shown to be a dominant strategy [44], while in other Chinese-based studies, *Hp* had at least a 99% probability of being cost-effective compared to no screening at a WTP of USD 31,315/QALY (2020 USD) from a healthcare provider perspective [36] and a WTP of CNY 80,976/QALY (perspective not reported) [2].

In other studies that considered primary intervention based on a combination of risk factors (including *Hp* infection, serum pepsinogen levels, G-17 levels, sex, and age), the intervention strategy was also proven to be cost-effective. These interventions include NGCS and GCRSS in China and the “ABC method” in Japan [2,30,31,32]. NGCS was a dominant strategy compared with no screening [2] and had an 86.3% probability of being cost-effective when screening started at age 40 years compared with no screening at a WTP of USD 17,922/QALY (2021 USD) from a societal perspective [30]. GCRSS was cost-effective with ICER ranging between USD 10,315 and 27,446/QALY at a WTP of USD 37,655/QALY from a healthcare system perspective [31]. ABC was also a dominant strategy with a 99.7% probability of being cost-effective at a WTP of USD 10,000/QALY compared to no screening from a healthcare payer perspective [32].

Meanwhile, for studies that compared endoscopic screening to no screening, the former was consistently the preferred strategy [13,25,29,34,38,40,45]. Biennial endoscopy had a 98% and a 100% probability of being cost-effective in China and Japan, respectively, from a healthcare system perspective compared to no screening [13,38]. Triennial screening was preferred 92.6% of the time compared with no screening among persons 50–75 years old (ICER: USD 45,665/QALY at a WTP of USD 50,000/QALY from a societal perspective [39]. For South Korea, at a WTP of USD 19,162 (2008 USD), endoscopic screening was cost-effective (ICER: USD 4820/QALY for males and USD 6073/QALY for females) from a societal perspective [34]. Similarly, in Singapore, screening was cost-effective (ICER: USD 26,836/QALY) at a WTP of USD 28,000 (2003 USD) from a societal perspective [45].

Among studies that focused on Western countries, two (one US-based study and one Portugal-based study) found that endoscopic GC screening performed during a colonoscopy was cost-effective compared to no screening (ICER: USD 74,329/QALY (2015 USD) at a WTP of USD 100,000/QALY, and ICER: EUR 30,908/QALY (2016 EUR) at a WTP of EUR 37,000/QALY [15,43]. An earlier US-based study, however, did not find this intervention to be cost-effective at a WTP of USD 50,000/QALY from a third-party payer perspective [46]. When considering only endoscopic screening, studies demonstrated that this strategy was cost-effective mainly for high-risk ethnic groups (Hispanics, non-Hispanic blacks, and Asians) in the USA (ICER: USD 71,451–USD 80,278/QALY (2015 USD) at a WTP of USD 100,000/QALY [15,42]. Screening using serum pepsinogen was only cost-effective among current and former smokers and not so for the general population in the USA compared to no screening [12]. This strategy had a 97% probability of being cost-effective at a WTP of USD 100,000/QALY from a societal perspective [12]. However, in a study based on a Mexican population, both serum pepsinogen and endoscopic screening were cost-effective (ICER: USD 1590/QALY and USD 129/QALY, respectively, at a WTP of USD 9000/QALY from a public healthcare perspective) [35].

Although the country settings of the selected studies varied widely across the world, most studies focused on GC cancer screening in China (*n* = 9). This was followed by Japan (*n* = 7), USA (*n* = 6), Republic of Korea (*n* = 3), and Singapore (*n* = 3). There was also one study that evaluated screening in Mexico [35] and another in Portugal [43], while another compared screening across countries in Asia, Europe, Africa, and South America [47].

The majority of these studies targeted high-risk populations within their respective jurisdictions including Japan, Republic of Korea, and regions within China that have higher incidence of GC compared to the general population. Studies based on Singapore, Mexico, and Portugal were assumed to have intermediate risk [35,40,41,43,45]. For the Singaporean studies, in particular, focus was on the Chinese subpopulation because of the higher risk of GC among that group [40,41,45]. Meanwhile, all the US-based studies proposed an average-risk general population with elevated risk among selected sub-population groups like, Asian Americans, non-Hispanic white, non-Hispanic blacks, and Hispanics [12,15,26,42,46,48]. See Table 1, Table 2 and Table 3.

### 3.3. Study Type and Model Parameters

Two types of cost-effectiveness studies were identified from the literature search: prospective observational cost-effectiveness studies and model-based cost-effectiveness studies. Five studies were observational in nature [25,28,29,49,50], while the remaining 26 studies presented model-based simulations (25 studies were state transition Markov models and 1 was a decision tree).

Regardless of the modeling approach adopted, the general consensus among the studies reviewed is that GC screening is cost-effective compared with no screening. Among the observational studies, focus was on high-risk populations in China and Republic of Korea [25,28,29,49,50]. GC (endoscopic) screening was cost-effective in Republic of Korea (ICER: KRW 119,099,000–17,870,000 KW/survival (WTP not reported) [29], and ICER: USD 20,309/LYS at a WTP of USD 20,565/LYS) [25]. Meanwhile, Wei et al. (2011) demonstrated that GC screening using endoscopy had a benefit–cost ratio ranging from 4.49 to 10.37 in Linzhou, China [28]. The other Chinese-based observational studies also reported that a comprehensive intervention inclusive of an epidemiology survey, serum pepsinogen testing, endoscopy, and a pathological examination with a positive identification was cost-effective (ICER: USD 459/QALY [49] and ICER: CNY 1370/QALY [50] (WTP not reported in both studies). With respect to model-based studies, the results are also consistent and can be gleaned from previous Section 3.1 and Section 3.2.

With observational studies, the cost-effectiveness of an actual population-based screening program was investigated; meanwhile, with model-based studies, hypothetical cohort groups were examined. Moreover, model-based studies implemented state transition models, typically of one-year cycle length, to investigate the impact of screening intervention on disease progression, clinical outcomes, and cost.

The state transition models adopted in most studies were based on Correa’s Cascade [20], which maps the stages towards the development of gastric cancer starting from normal mucosa to chronic gastritis, atrophic gastritis, and intestinal metaplasia and then to dysplasia. Some studies further captured the various stages of gastric carcinoma from stages I to IV in their proposed models and, in some cases, distinguished between clinical and preclinical health states based on whether a clinical diagnosis was established [2,12,13,14,15,30,31,35,39,42,44]. Yet, other studies focused on *Hp* screening and eradication and then transitioned cohort members to GC stages without accounting for the other precancerous health stages [32,33,36,37,38,48]. Another group of studies focused solely on GC screening and the related cascading GC health states, not accounting for precancerous stages [34,40,43,47]. Meanwhile, Gupta et al. (2011) modeled the precancerous states that precede GC but had no explicit account of the stages of GC [46].

For observational studies, the time horizon ranged between 2 and 10 years, while for model-based studies, the time horizon ranged from 15 years to a lifetime. Moreover, among the model-based studies, 21 used a time horizon of at least 25 years. The starting age for screening was generally either 40 or 50 years and continued until at least age 69 years in most cases, or death, whichever occurred first.

Screening compliance rates also differed across studies, ranging from 13% to 100%. Studies that adopted a rate less than 100% either reflected current screening rates or a minimum ideal rate to establish cost-effectiveness. Meanwhile, those with full screening compliance sought to express the maximum potential benefit of the screening program [2,30,31,41,45,46,47,48,49,50].

The input parameters were sourced mainly from the existing literature; clinical databases and registries; clinical trials; perspective data from the study population; health insurance databases; and the National Cancer Institute—Surveillance, Epidemiology, and End Results Program. Discount rates were either 3% or 5%.

While most studies focused on populations at higher risk of GC, fewer studies investigated populations at average and intermediate risk, where risk was primarily measured by the presentation of *Hp* infection, gastric atrophy, and/or intestinal metaplasia. Other risk factors considered were familial exposure to *Hp* and smoking history. See Table 1, Table 2 and Table 3.

### 3.4. Willingness-to-Pay Thresholds and Sensitive Variables

In terms of the willingness-to-pay (WTP) threshold, studies either used the World Health Organization’s recommendation of less than three time the annual national gross domestic product (GDP) per capita or a typical threshold level, such as USD 50,000/QALY or USD 100,000/QALY, or a level recommended within a particular local setting [51,52]. See Table 1.

For some models, ICER was sensitive to transition probabilities, prevalence of disease and risk factors, distribution of cancer at screening, screening age, utility scores, discount rate, cost of screening and cost of follow-up procedures, compliance with treatment, and sensitivity and specificity of the screening test. Notwithstanding, most models were stable and ICER did not vary significantly when parameters were allowed to change independently. See Table 3.

### 3.5. Results on Quality of Reporting

The results of the 28-item CHEERS 2022 [24] checklist for the 31 selected studies are presented in Supplementary Table S1 in the ESM. Based on the CHEERS checklist, the quality of reporting was generally consistent among the selected articles, although a number of studies pre-dated the updated CHEERS 2022 [24] reporting guidelines and so some checklist items were not included. For example, of the 28 items, no study reported on criteria 21 and 25, which address patient and other study stakeholder engagements. Although some studies mentioned that the CHEERS checklist was used for reporting, albeit the previous version (CHEERS 2013) [53], many studies did not provide this list either in the main text or Supplementary Materials. Also, some studies did not report conflicts of interest and source of funding. See Table 3 and Supplementary Table S1 in the ESM.

## 4. Discussion

This systematic review revealed that screening for GC is always preferred to no screening, as demonstrated by cost-effectiveness analyses. Notwithstanding this, there was some variability in the results, mainly because the intervention strategies differed notably from one study to the next. These strategies included different combinations of primary and secondary GC screening tests, and follow-up treatments. Cost-effectiveness was also influenced by the incidence of GC in a given location; the type of screening intervention; and the prevalence of risk factors, mainly *Hp* infections. Overall, there was general agreement that GC screening is likely to be cost-effective in countries or regions or among high-risk populations where the incidence of disease is relatively high and the cost of screening is relatively low. This sentiment was expressed by Ascherman and Hur (2021) in an economic evaluation of endoscopic GC screening among Brazil, France, Japan, Nigeria, and the United States [47].

As a primary intervention, *Hp* screening and treatment is potentially cost-effective in high-risk regions. Lansdorp-Vogelaar et al. (2021) had a similar conclusion in a review of model-based studies that investigated the cost-effectiveness of GC screening and surveillance in Western countries [54]. Meanwhile, secondary interventions that involve endoscopic screening are also cost-effective, but they only address disease malignancies rather than precancerous conditions. For countries with average to intermediate risk of GC, endoscopic screening of subpopulations with elevated risk of disease was also cost-effective. This was evident predominantly in the Chinese diaspora in other Asian countries and in the USA. Other high-risk diaspora groups include Hispanics and non-Hispanic blacks in the USA [42].

The value of primary intervention is that it allows for the detection and treatment of precancerous conditions, and it stymies, if not prevents, progression to malignancy and, ultimately, death [6,7,8]. It also helps to identify those at varying degrees of gastric cancer risk based on serological findings, *Hp* infection, smoking history, age, and ethnicity. This facilitates a more tailored approach with respect to surveillance and treatment of each case [40]. Primary intervention strategies including screening, early detection, and surveillance are also practical given the window of opportunity between gastric atrophy and tumor presentation [42,55,56].

Guidelines for primary intervention typically recommend mass *Hp* screening and eradication therapy (in asymptomatic persons) for the prevention of GC in populations at elevated risk of developing GC [22,57,58,59]. The full benefits of *Hp* eradication strategies are only realized in the long-run, manifesting in fewer cases of GS, fewer related deaths, and cost-savings [31,60]. This long-run effect also accounts for the disparity in results among studies with longer versus shorter time horizons. It is also a critical factor to consider in deciding on *Hp* intervention across different populations, especially since *Hp* screening has higher sensitivity and specificity than UGI and endoscopy [33].

Meanwhile, according to one meta-analysis, endoscopic screening programs have the potential to reduce GC deaths by 40% and is therefore considered the golden standard of GC screening and detection [47,61]. However, this tool is relatively invasive and may be associated with lower compliance rates [40]. Nonetheless, the combination of *Hp* screening and eradication and follow-up endoscopy is an effective strategy for addressing the burdens associated with GC [37,62].

The findings from the studies highlighted in this systematic review are instructive to policy makers in deciding on GC intervention strategies for their respective jurisdictions. These findings suggest that interventions for GC are best tailored to a specific country setting and local context related to the incidence of disease, the prevalence of risk factors, and the cost and benefit of screening. A similar view was echoed by the Working Group of the International Agency for Research on Cancer (IARC) in its recommendation for countries, particularly those with a high incidence of GC, to assess their need for a gastric cancer program in light of the potential benefits and costs of such a program over time [22]. The Working Group further advised that in developing a national *Hp* screening and treatment program, a country should account for factors such as local disease incidence and distribution, cost, effectiveness, and other competing health challenges, while ensuring that interventions are evidence-based [17]. Furthermore, the outcome of an economic evaluation of a cancer screening program, especially for gastric cancer, hinges largely on the quality of parameter estimates, model specification, the diagnostic performance of screening tools, and the level of risk of cancer among the target population.

The level of cancer risk is important because it influences cancer incidence rates. As *Hp* infection is the primary risk factor for GC, particularly of the non-cardia type, populations with the highest infection rates tend to also have a greater incidence of GC [2,12,15,16,17]. This is true for Asian countries like China and Japan, as well as their diaspora, where *Hp* infection and GC are more prevalent [1]. Furthermore, although the number of cases of GC has lowered over the years, the projected incident rate is expected to remain high in the future [63]. Notwithstanding, the risk of GC is expected to decrease over time in countries at higher risk with successful *Hp* eradication strategies and also among high-risk diaspora populations in the absence of other environmental and extrinsic risk factors [42,64]. This reality also has implications for the cost-effectiveness of screening strategies as they are expected to erode after each successive generation [42,64].

Countries at higher risk of GC have an age-standardized rate (ASR) of ≥20 per 100,000 and are ideal candidates for nationwide screening programs [43,65]. In 2020, the countries with the highest risk, as measured by ASR, included Mongolia (32.5), Japan (31.6), the Republic of Korea (27.9), Tajikistan (23.4), China (20.6), and Kyrgyzstan (19.7) [1]. National gastric cancer screening programs using radiographic screening and endoscopic examination currently exist in Japan and Republic of Korea, while in China, screening programs have emerged predominantly in regions where the incidence of disease is particularly high [13]. Relatedly, Japan and Republic of Korea have experienced declines in the incidence and burden of GC linked to their respective national screening programs, while China continues to grapple with these challenges due in part to an aging population, limited resources given the scale of intervention needed to implement a national screening program, and notable regional diversity [44,66]. These factors may account for the fact that <10% of diagnosed cases are at the early stage of the disease in China, while in Japan and Republic of Korea, early-stage diagnoses account for 50–70% of all diagnosed cases [9,10,16,67]. As a comparison, in the USA, where no screening program exists, 75% of diagnosed cases occur at later stages of disease [42].

For countries with low and intermediate risk of GC (ASR < 10 per 100,000), population-wide screening is nonexistent because it is not cost-effective, and what is often recommended is surveillance and treatment of those with precancerous conditions like *H pylori* infections, atrophic gastritis, and intestinal metaplasia [43,58,68,69]. In fact, in the USA, researchers found that among 50-year-old men, endoscopic mucosal resection (EMR) with endoscopic surveillance either every 1, 5, or 10 years for gastric dysplasia was cost-effective (ICER: USD 18,600, USD 20,900, and USD 39,800/QALY, respectively, at a WTP: USD 100,000/QALY, (2007 USD) from a societal perspective [70]. Although this intervention potentially reduces the risk of developing GC by at least 60%, its cost-effectiveness was less favorable on less advanced precancerous condition like intestinal metaplasia, with the possible exception of migrant populations from high-risk countries [70].

One meta-analysis demonstrated that even though immigrants may move from a country with a high incidence of GC to one with low incidence, they still maintain an inherent elevated risk of developing GC [71]. Building on this, Shah et al. recounted the fact that the incidence of GC is notably greater in certain minority groups in the US, namely Hispanic and non-Hispanic blacks, and some Asian American groups, which have incidence rates two and six times that of non-Hispanic whites, respectively [15]. Further investigation found that for these minority groups, a one-time GC screening using esophagogastroduodenoscopy/upper GI endoscopy (EGD) during a colonoscopy, with follow-up EGDs only if indicated, is cost-effective [42].

One region where further research is needed to confirm the cost-effectiveness of gastric cancer screening is Africa. While the continent has the highest prevalence of *Hp* infections globally, with about 70% of the population infected, the incidence of gastric adenocarcinoma remains relatively low compared to other regions (the age-standardized incidence rates per 100,000 of GC in 2022 for Africa, North America, Oceania, Europe, Latin America and the Caribbean, and Asia were 4, 4.1, 5.5, 7.9, 8.5, and 11, respectively [1])—a phenomenon called the “African Enigma” [72,73]. The enigma is prefaced on the fact that the clinical presentation of patients in Africa with gastrointestinal conditions, particularly gastric atrophy, stemming from *Hp* infection is similar to that of patients in Western and Asian countries; however, progression to GC is less common [72,74,75]. Researchers proffered several reasons for this epidemiological puzzle. Some credit under-resourced health systems and the absence of cancer registries for under-reporting of cancer cases [76]. Others argue that the virulence of the strain of the *Hp* bacteria most prevalent in Africa (HpAfrica2) is less severe than those found in other regions, lacking the Cag Pathogenicity Island, which is linked to the development of GC [77]. Relatedly, it has also been argued that the development of GC from *Hp* infections can be stymied in cases of co-parasitic infections that may alter the progression of disease by delaying atrophic gastritis [78]. The impact of a diet consisting of fresh fruits and vegetables, which helps with reducing the risk of GC, has also been credited to be responsible for the enigma [79].

This systematic review has limitations. The main limitation is the exclusion of studies not written in English. The authors recognize that there are a number of economic evaluation studies written in languages other than English that were not included in this research. However, we anticipate that the conclusion of this review would not change even if those studies were included. We also acknowledge that the treatment strategies for *Hp* infection and GC would impact the cost-effectiveness of screening. However, these various strategies were not explicitly accounted for in this analysis. We are of the opinion that this analysis is beyond the scope of this review.

## 5. Conclusions

Gastric cancer (GC) screening can be cost-effective, but cost-effectiveness is dependent on context specific factors, including geographical location, the prevalence of GC in the local population, and the screening tool adopted. There is some benefit to targeting high-risk population groups like Asians and their diaspora for screening, as well as current and former smokers. Primary interventions (including *Hp* screening and screening based on multiple risk factors) and secondary intervention (endoscopic screening) were found to be cost-effective in high-risk regions compared with no screening. However, primary interventions were preferred to secondary interventions when both types of strategies were compared. In studies that focused on countries with intermediate- and average-risk regions, population-wide screening was not cost-effective, but screening was cost-effective when special population groups were targeted, e.g., Hispanics and non-Hispanic blacks in the USA, the Asian diaspora in the USA and Singapore, as well as current and former smokers.

## Data Availability

The protocol for this systematic review is available in the International Prospective Register of systematic reviews, PROSPERO, under registration number CRD42023467167: https://www.crd.york.ac.uk/prospero/, accessed on 14 October 2023. The search strategy and results of the 28-item CHEERS 2022 checklist for the 31 selected studies are available in the Supplementary Files while the results of the search strategy are included in this manuscript.

## Supplementary Materials

The following supporting information can be downloaded at: https://www.mdpi.com/article/10.3390/cancers16132353/s1, Table S1: CHEER checklist; File S1: ESM Search strategy.
