# Supplementary material for: A Systematic Review of Cost-Effectiveness Studies on Gastric Cancer Screening
Source: Cancers (Basel). 2024 Jun 27;16(13):2353. doi: 10.3390/cancers16132353 (PMC11240801; doi:10.3390/cancers16132353)
Supplement: Supplementary file 1 [file cancers-16-02353-s001.zip › File S1 ESM Search strategy.pdf]

A systematic review of cost-effectiveness studies on gastric cancer screening

Diedron Lewis[1], Laura Jimenez[2], Manel Haj Mansour[3], Susan Horton[4], William W L Wong[1]

[1]School of Pharmacy, University of Waterloo, Waterloo, ON, Canada
[2] Department of Community Health and Epidemiology, Dalhousie University, Halifax, NS, Canada
[3] Aga Khan University Hospital, Nairobi, Kenya
[4] School of Public Health Sciences, University of Waterloo, Waterloo, ON, Canada

ESM: search strategy

PubMed query

Category	Search terms
#1	(Esophageal Neoplasms [mesh] OR Stomach Neoplasms [mesh] OR Liver Neoplasms [mesh] OR Pancreatic Neoplasms [mesh] OR ((liver[tw] OR esophageal[tw] OR stomach[tw] OR pancreatic[tw]) AND (cancer[tw] OR carcinoma[tw] OR tumor[tw] OR tumour[tw])) OR Liver cancer[tw] OR esophageal cancer[tw] OR oesophageal cancer[tw] OR pancreatic cancer[tw] OR stomach cancer[tw] OR gastric cancer[tw] OR Cirrhosis[tw] OR Hepatocellular[tw] OR Liver Neoplasms[mesh] OR Liver Cirrhosis[mesh] OR Hepatitis C[mesh] OR Hepatoma[tw] OR Hepatoblastoma[tw] OR Tumour[tw] OR adenocarcinoma[tw] OR squamous[tw])
#2	screening[tw] OR Detection[tw] OR Mass screening[mesh] OR Early detection of cancer[mesh])
#3	Cost benefit analysis[mesh] OR Cost analysis[mesh:noexp] OR Economics[subheading] OR Cost effectiveness[ti] OR Cost benefits[ti] OR Cost analysis[ti] OR Economic evaluations[ti] OR Cost utility[ti] OR cost [ti] OR economic*[ti])
#1 AND #2 AND #3	(Esophageal Neoplasms [mesh] OR Stomach Neoplasms [mesh] OR Liver Neoplasms [mesh] OR Pancreatic Neoplasms [mesh] OR ((liver[tw] OR esophageal[tw] OR stomach[tw] OR pancreatic[tw]) AND (cancer[tw] OR carcinoma[tw] OR tumor[tw] OR tumour[tw])) OR Liver cancer[tw] OR esophageal cancer[tw] OR oesophageal

	cancer[tw] OR pancreatic cancer[tw] OR stomach cancer[tw] OR gastric cancer[tw] OR Cirrhosis[tw] OR Hepatocellular[tw] OR Liver Neoplasms[mesh] OR Liver Cirrhosis[mesh] OR Hepatitis C[mesh] OR Hepatoma[tw] OR Hepatoblastoma[tw] OR Tumour[tw] OR adenocarcinoma[tw] OR squamous[tw]) AND (screening[tw] OR Detection[tw] OR Mass screening[mesh] OR Early detection of cancer[mesh]) AND (Cost benefit analysis[mesh] OR Cost analysis[mesh:noexp] OR Economics[subheading] OR Cost effectiveness[ti] OR Cost benefits[ti] OR Cost analysis[ti] OR Economic evaluations[ti] OR Cost utility[ti] OR cost [ti] OR economic*[ti])

#5	Search: **#2 AND #3 AND #4**
#4	Search: **(Cost benefit analysis[mesh] OR Cost analysis[mesh:noexp] OR Economics[subheading] OR Cost effectiveness[ti] OR Cost benefits[ti] OR Cost analysis[ti] OR Economic evaluations[ti] OR Cost utility[ti] OR cost [ti] OR economic*[ti])**
#3	Search: **screening[tw] OR Detection[tw] OR Mass screening[mesh] OR Early detection of cancer[mesh]**

#2	Search: **(Esophageal Neoplasms [mesh] OR Stomach Neoplasms [mesh] OR Liver Neoplasms [mesh] OR Pancreatic Neoplasms [mesh] OR ((liver[tw] OR esophageal[tw] OR stomach[tw] OR pancreatic[tw]) AND (cancer[tw] OR carcinoma[tw] OR tumor[tw] OR tumour[tw])) OR Liver cancer[tw] OR esophageal cancer[tw] OR oesophageal cancer[tw] OR pancreatic cancer[tw] OR stomach cancer[tw] OR gastric cancer[tw] OR Cirrhosis[tw] OR Hepatocellular[tw] OR Liver Neoplasms[mesh] OR Liver Cirrhosis[mesh] OR Hepatitis C[mesh] OR Hepatoma[tw] OR Hepatoblastoma[tw] OR Tumour[tw] OR adenocarcinoma[tw] OR squamous[tw])**

Scopus query

Category	Search terms
#1	((TITLE-ABS-KEY ("Esophageal Neoplasms" OR "Stomach Neoplasms" OR "Liver Neoplasms" OR "Pancreatic Neoplasms") OR TITLE-ABS-KEY (("liver" OR "esophageal" OR "Hepatocellular" OR "gastric" OR "stomach" OR "pancreatic" OR "Hepatoma" OR "Hepatitis C" OR "Cirrhosis") W/3 ("cancer" OR "carcinoma" OR "tumor" OR "tumour" OR "adenocarcinoma" OR "squamous")) OR TITLE-ABS-KEY ("Hepatoblastoma")))
#2	(TITLE-ABS-KEY ("screening" OR "Detection" OR "Mass screening" OR "Early detection of cancer"))
#3	(TITLE-ABS-KEY ("Cost benefit*" OR "Cost analysis" OR "Cost effectiveness" OR "Economic evaluations" OR "Cost utility"))
#1 and #2 and #3	((TITLE-ABS-KEY ("Esophageal Neoplasms" OR "Stomach Neoplasms" OR "Liver Neoplasms" OR "Pancreatic Neoplasms") OR TITLE-ABS-KEY (("liver" OR "esophageal" OR "Hepatocellular" OR "gastric" OR "stomach" OR "pancreatic" OR "Hepatoma" OR "Hepatitis C" OR "Cirrhosis") W/3 ("cancer" OR "carcinoma" OR "tumor" OR "tumour" OR "adenocarcinoma" OR "squamous")) OR TITLE-ABS-KEY ("Hepatoblastoma"))) AND (TITLE-ABS-KEY ("screening" OR "Detection" OR "Mass screening" OR "Early detection of cancer")) AND (TITLE-ABS-KEY ("Cost benefit*" OR "Cost analysis" OR "Cost effectiveness" OR "Economic evaluations" OR "Cost utility"))
